# Exploring the Impact of Neurophysiotherapy in Managing Leukoencephalopathy Challenges: A Case Report

**DOI:** 10.7759/cureus.56452

**Published:** 2024-03-19

**Authors:** Ghanishtha C Burile, Nikita H Seth, Raghumahanti Raghuveer

**Affiliations:** 1 Neurophysiotherapy, Ravi Nair Physiotherapy College, Datta Meghe Institute of Higher Education and Research, Wardha, IND

**Keywords:** physiotherapy rehabilitation, posterior occipital and parietal lobes, microangiopathies, colony-stimulating factor 1 receptor mutations, posterior reversible encephalopathy syndrome, white matter, leukoencephalopathy

## Abstract

Leukoencephalopathy (LE), characterized by structural changes affecting cerebral white matter, presents a complex clinical picture with diverse etiologies. This case report details the presentation, clinical findings, and physiotherapy management of a 32-year-old female with colony-stimulating factor 1 receptor (CSF1R)-related leukoencephalopathy and a history of diabetes and hypertension. She suddenly stopped her medications, which led to the worsening of her condition. She presented with symptoms of headache, slurred speech, visual disturbances, cognitive impairment, and impaired balance and coordination, due to which her activities of daily living were affected. The symptoms highlighted the challenges and multidisciplinary approach required for its management. The patient exhibited neurological deficits, cognitive decline, and abnormal reflexes, with magnetic resonance imaging (MRI) revealing white matter abnormalities. Outcome measures demonstrated significant improvements in cognitive and functional abilities, emphasizing the effectiveness of tailored rehabilitation in managing the complexities of colony-stimulating factor 1 receptor-related leukoencephalopathy. A six-week physiotherapy rehabilitation program addressed various domains, including strength training, task-specific exercises, errorless learning, facial muscle retraining, balance exercises, visual restoration therapy, and mobility training. All these interventions effectively improved her functional capacity and made the patient independent in performing activities of daily living.

## Introduction

A structural change primarily affecting myelin in the cerebral white matter is known as leukoencephalopathy (LE). Toxic leukoencephalopathy can be caused by exposure to a variety of substances, such as medicinal agents, illicit narcotics, environmental pollutants, and cranial irradiation. The white matter tracts in charge of higher cerebral function are principally affected by this illness, which can lead to a variety of clinical symptoms such as dementia, inattention, forgetfulness, personality changes, and, in rare circumstances, death [1]. Eight cases of "encephalitis subcortical progressive," in which white matter abnormalities were evident and resulted in vascular alterations, were reported by Binswanger in 1894. This was clinically seen as a progressive mental decline accompanied by "apoplectic attacks." Since then, leukoencephalopathy (LE) in older people with chronic hypertension has been referred to as Binswanger's illness. Progressive arteriosclerosis that affects the deep medullary branches in the cerebral hemisphere white matter can lead to leukoencephalopathy [2]. Arteriosclerosis in the cerebral small arteries, along with thickening and splitting of the internal elastic lamina, loss of vascular smooth muscle cells, and hyaline degeneration of the media, is the characteristic pathological feature of cerebral autosomal recessive arteriopathy with subcortical infarcts and leukoencephalopathy (CARASIL) [3]. Neuropathologically, neuronal axons, blood vessel components, and glial cells such as oligodendrocytes, astrocytes, and microglia are all involved in different combinations in adult-onset leukoencephalopathies [4]. The white matter vasogenic edema that characterizes posterior reversible encephalopathy syndrome (PRES) is a clinical-radiological condition that mostly affects the brain's posterior occipital and parietal lobes. Headaches, convulsions, changed mental status, and visual loss are some more signs and symptoms of PRES. It can occur in patients with immunosuppressed conditions, hypertension, or renal insufficiency [5].

A hereditary leukoencephalopathy should be considered if a patient with dementia exhibits neurological symptoms such as pyramidal signs (spastic paraparesis and hyperreflexia), ataxia, extrapyramidal indications, bulbar dysfunction, peripheral neuropathy, or autonomic dysfunction [6]. Stroke-related perceptual effects, such as inattention and impairment in cortical visual processing, affected one-fifth of the referred patients [7]. It is unclear why colony-stimulating factor 1 receptor (CSF1R) mutations cause white matter degeneration in a specific way. Because CSF1R is mostly expressed in microglia, CSF1R-related leukoencephalopathy is a type of primary microgliopathies in which microglia play a crucial and fundamental role in the pathophysiology of these diseases [8]. CSF1R-related leukoencephalopathy is incurable, just as the majority of other hereditary leukoencephalopathies. Symptomatic therapy, such as muscle relaxants for spasticity, antidepressants for depression, and, if acceptable, antiepileptic medications for epilepsy, should be provided even though the expected effect is minimal [9]. According to one study, CSF1R-related leukoencephalopathy accounts for 10% of adult-onset leukoencephalopathies and may be the most prevalent kind [10]. For patients in whom myelination is affected, rehabilitation is an integral component of the care, and it should be planned as soon as the patient becomes able to participate in rehabilitation therapies. A good neurological assessment should be done to plan rehabilitation, the objective of which would be a goal-oriented program that focuses on reducing symptoms and improving functional capacity [11].

## Case presentation

We present the case of a 32-year-old female patient who was fine one year ago. Following the delivery of her first child, she experienced a cerebrovascular event resulting in weakness in her left upper and lower limbs. Again, six months after this incident, she began to notice weakness in her right upper and lower limbs, followed by difficulty performing daily activities. Concerned about these symptoms, she was brought to a tertiary care hospital. Upon evaluation, the patient reported a history of hypertension for eight years, for which she had been prescribed antihypertensive medication. However, she had discontinued her antihypertensive treatment. Following this discontinuation, she experienced a sudden onset of symptoms, including headache, weakness in her right upper and lower limbs, and a progressive deterioration in her ability to stand for prolonged periods, walk, remember, and communicate effectively. She also noted visual disturbances, speech impairments, and perceptual disturbances. In investigations, a magnetic resonance imaging (MRI) of the brain was done. A thorough neurological examination was conducted, assessing tone, gait, reflexes, and other relevant parameters. Subsequently, she was admitted to the neurology ward in the first week of December 2023. MRI of the brain was done, and the patient was diagnosed with cerebral autosomal dominant arteriopathy with subcortical infarcts and leukoencephalopathy (CADASIL). Immediately after the diagnosis of leukoencephalopathy, the patient was referred to physiotherapy. The primary goal of physiotherapy was to relieve the symptoms of weakness on the right side of the body, early fatigue, difficulty in standing for prolonged duration, and speech, visual, and perceptual disturbances and improve functional capacity for a better quality of life.

Clinical findings

Informed consent was taken before evaluation and treatment. A detailed neurological examination was done. On muscle tone examination according to the tone grading scale (TGS), it was 3+(hypertonia) in the right upper and lower limb; according to the Modified Ashworth scale (MAS), it was 1+ (slight increase in tone, catch/release and resistance through rest range of motion (ROM) 1/2 ROM). According to voluntary control grading (VCG), it was grade 2 (indicates half ROM in synergy or abnormal pattern in the right upper and lower limb). The sensory assessment was done with the help of monofilament. It indicates that all sensations were intact. Reflex assessment reveals hyperactive reflexes on the right side and normal reflexes on the left side (pre-intervention) (Table 1).

**Table 1:** Reflex examination (pre-intervention) ++: normal, +++: exaggerated

Reflexes	Right	Left
Biceps jerk	+++	++
Triceps jerk	+++	++
Supinator jerk	+++	++
Knee jerk	+++	++
Ankle jerk	++	++
Plantar reflex	Extended	Extended

Outcome measures

Assessment of all outcome measures was taken before the treatment. Significant improvement was seen post-intervention. Examination for voluntary control was taken, which was grade 4 right upper and lower limb (initial half range is performed in isolation and later half in pattern). Table 2 shows the assessment of pre- and post-intervention outcome measures.

**Table 2:** Outcome measures MoCA: Montreal Cognitive Assessment, FIM: Functional Independence Measure

Outcome measures	Pre-intervention	Post-intervention
MoCA	9/30 (severe cognitive impairment)	17/30 (moderate cognitive impairment)
Barthel Index	21 (severe dependency)	67 (moderate dependency)
FIM	2 (maximal assistance)	4 (minimal assistance)

Investigations

**Figure 1:**
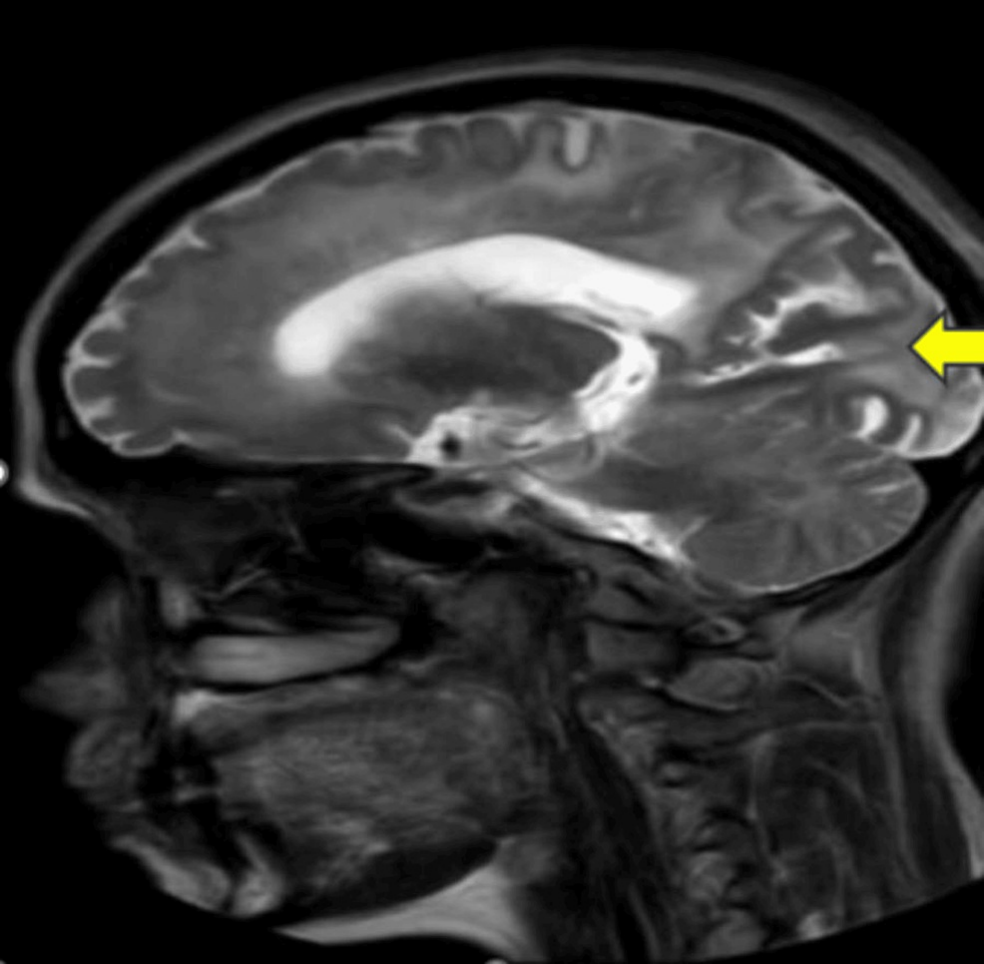
MRI of the brain revealed leukoencephalopathy (CADASIL) (yellow arrow) MRI: magnetic resonance imaging, CADASIL: cerebral autosomal dominant arteriopathy with subcortical infarcts and leukoencephalopathy

In investigations, an MRI of the brain was done. In Figure 1, an MRI of the brain revealed cerebral autosomal dominant arteriopathy with subcortical infarcts and leukoencephalopathy (CADASIL).

Physiotherapy management

A physiotherapy rehabilitation protocol was designed depending on the severity of symptoms and was progressed accordingly to improve the patient's functional capacity (Table 3). The rehabilitation program was designed for a duration of six weeks.

**Table 3:** Physiotherapy management Sources: [12-17] VRT: visual restoration therapy

Problem list	Goals	Interventions
To reduce weakness and improve functioning	To reduce weakness in mostly right side of the body in both upper and lower limbs	Strength training: task-specific training including reaching toward objects placed at certain distances, glass holding, bimanual tasks including catching and throwing of objects, and grasping the object while calculating. These activities produce modality-specific brain changes (angiogenesis in response to aerobic activity and synaptogenesis in response to motor skill training) and typically promote a task-specific approach.
To reduce symptoms such as memory loss, forgetfulness, and mild cognitive impairment	To reduce symptoms of retrograde amnesia and dementia	Errorless learning intervention: the term "errorless learning" describes the methodical titration of challenge level to ensure that the patient gains experience with increasing challenge and learns without having to rely on trial and error.
Difficulty in completing sentences while communicating	To reduce the symptoms of aphasia	Speech and language therapy for 30-60 minutes per day, two days a week.
Facial muscle weakness	To improve facial movements	Facial muscle retraining exercises, smiling exercises, facelift exercises, lip exercises, and exercises to strengthen the mandibular shapes.
Impaired balance and coordination	To improve static and dynamic balance (sitting and standing) and coordination, improve gait pattern, and reduce the risk of fall	In sitting: for static balance, weight shifts in sitting on a stable surface (10 repetitions × 2 sets); once static balance is achieved, it can be further progressed to dynamic balance in sitting with the help of a Swiss ball (unstable surface) (10 repetitions × 2 sets). In standing: for static balance, weight shifts in standing, tandem standing, and multidirectional reachouts once static balance is maintained; it can be progressed to an unstable surface such as walking on a soft mattress or walking on sand (10 repetitions × sets) to improve gait pattern; walking with the help of walker, initially starting with 1-2 rounds; walking with parallel bar support, initially 2-3 rounds and then progress depending upon patients' tolerance; walking around obstacles and walking over obstacles; and sideways walking.
Visual disturbances (spatial neglect)	To make the vision clear (poor eyesight and diplopia)	VRT: visual feedback has shown improvements in stance symmetry, vergence exercises for unilateral visual inattention, and bottom-up and top-down interventions.
Perceptual disturbances	To reduce symptoms of spatial neglect syndrome	Mirror therapy used to treat motor and perception problems and perceptual exercises such as pegboard activities or parquetry blocks and puzzles.
Urinary incontinence	To improve bladder control	Pelvic floor strengthening exercises, static abdomen, and sensory-motor biofeedback device.

**Figure 2:**
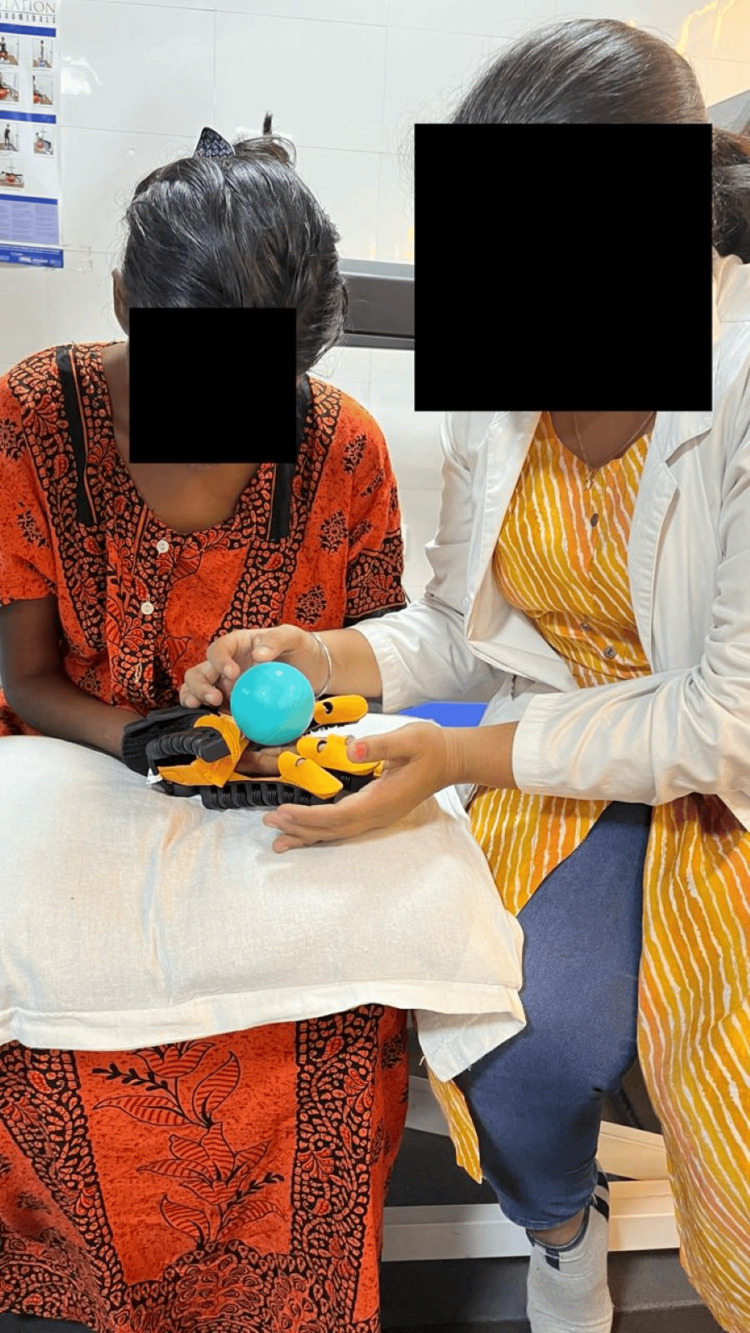
Hand rehabilitation using a robotic glove

In Figure 2, the patient was given hand rehabilitation using a robotic glove.

**Figure 3:**
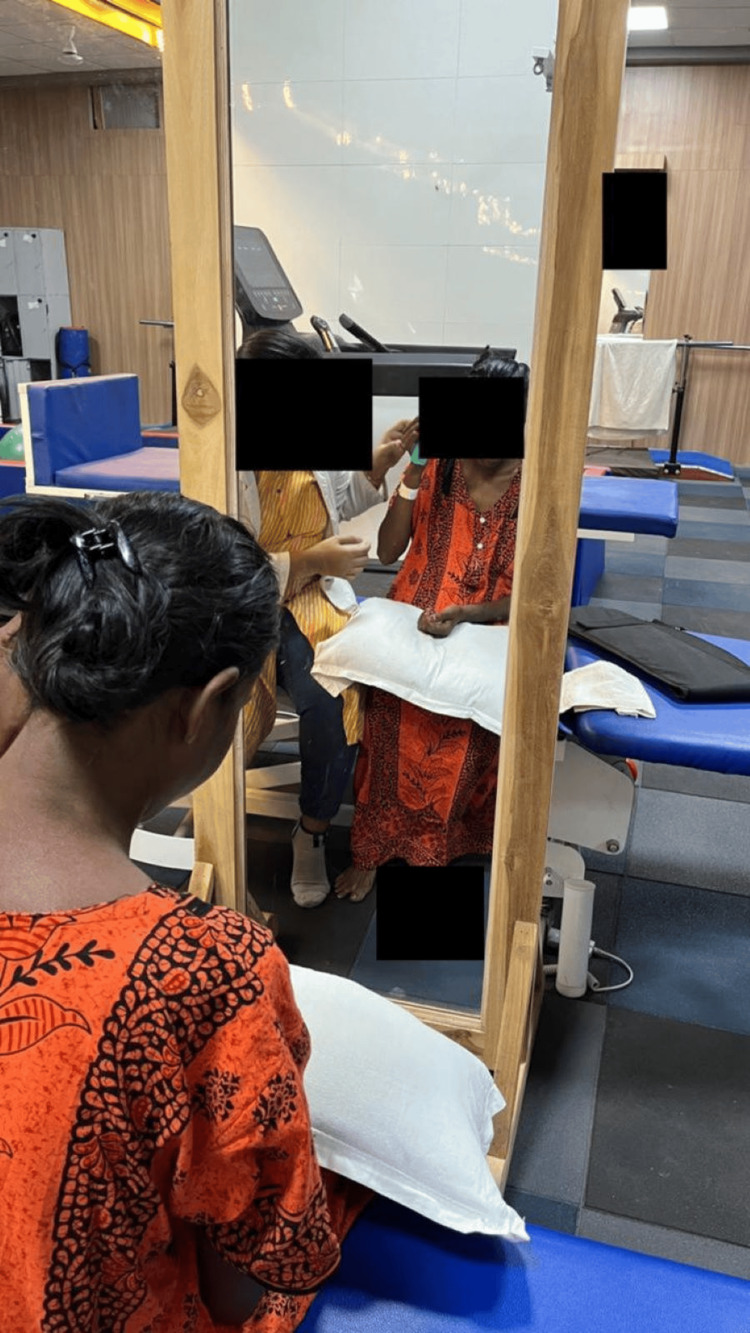
Training for basic activities of daily living using a mirror to provide visual biofeedback

 Training was done for basic activities of daily living using a mirror to provide visual biofeedback (Figure 3).

**Figure 4:**
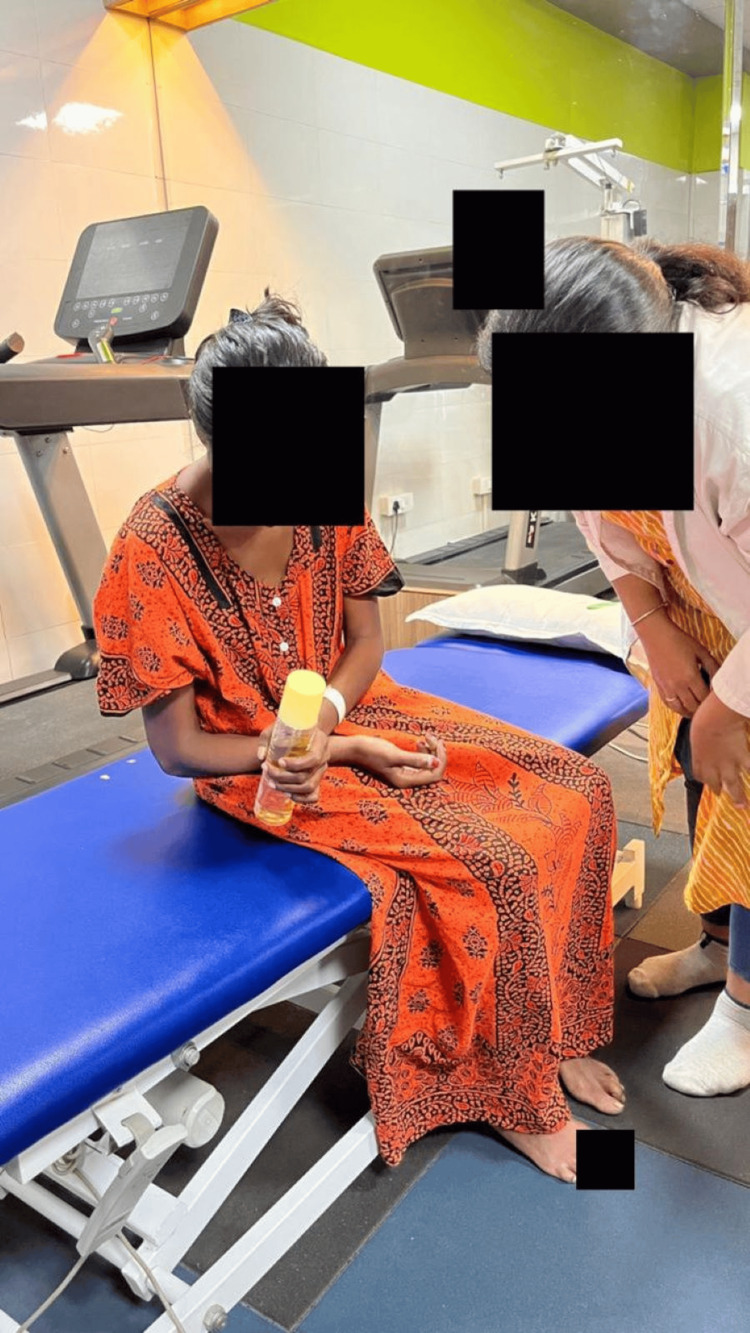
Task-oriented approach for training upper limb mobility

A task-oriented approach was used for training upper limb mobility (Figure 4).

**Figure 5:**
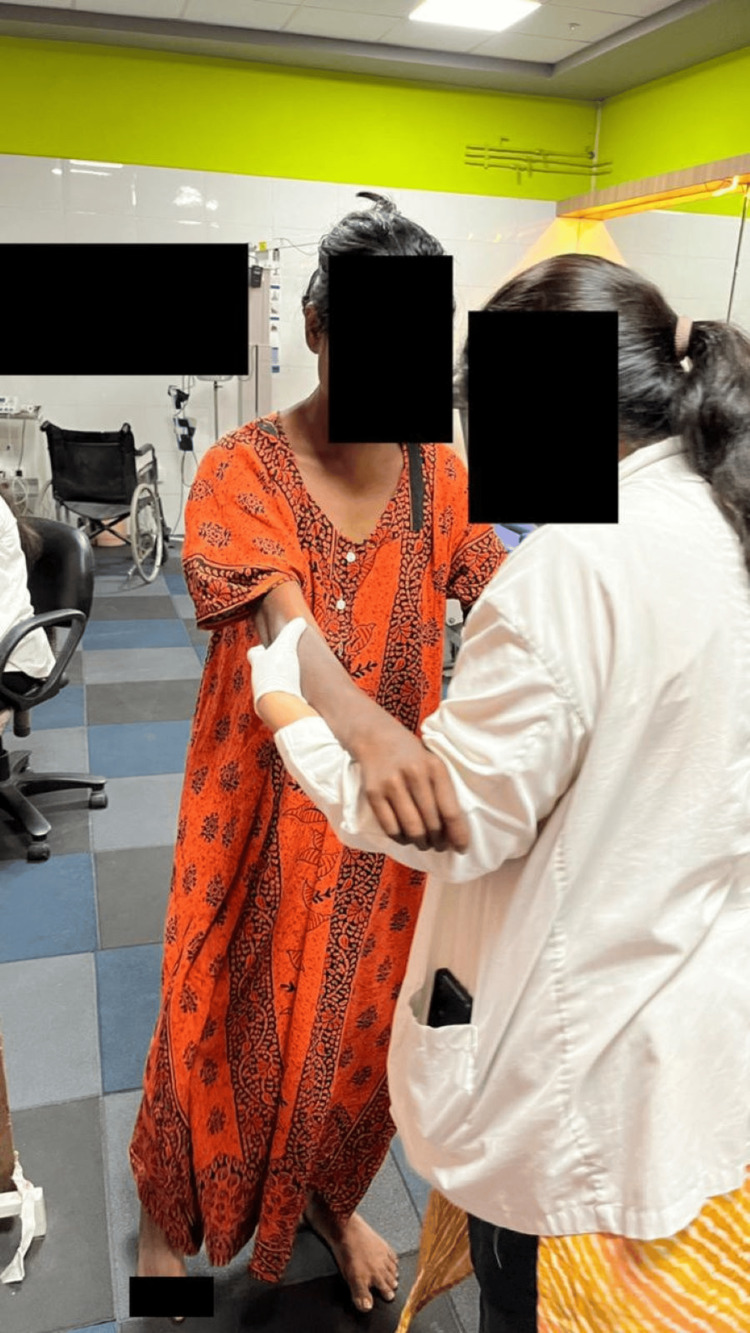
Gait training with the assistance of the therapist

Gait training was conducted with the assistance of the therapist (Figure 5).

## Discussion

The condition known as reversible posterior leukoencephalopathy syndrome is characterized by radiological abnormalities that mostly affect the parieto-occipital lobe white and gray matter. The sudden increase in blood pressure that disrupts the autoregulatory mechanisms of the central nervous system vasculature may be the cause of reversible posterior leukoencephalopathy syndrome, which is linked to hypertension. This disturbance can lead to focal fluid transudation and petechial hemorrhages, as well as the establishment of areas of vasodilation and constriction and the collapse of the blood-brain barrier [18,19]. According to a study conducted by Liao et al. [20], patients with intracerebral hemorrhage who had cerebral autosomal dominant arteriopathy with subcortical infarcts and leukoencephalopathy (CADASIL) had a much higher mortality rate (28.6% versus 8.7%) and a 2.17-fold increased risk of recurrent stroke; in clinical care and prognosis prediction, identifying patients with CADASIL who are at risk of hemorrhagic stroke is critical [20,21]. Delayed post-hypoxic leukoencephalopathy (DPHL) is a unique clinical entity that presents with cognitive impairment days to weeks after an episode of acute hypoxic brain injury [22]. Plum et al. [23] documented multiple cases of DPHL associated with complications from surgical anesthesia, cardiac arrest, or CO poisoning.

In the above case, a 32-year-old female with a history of hypertension and diabetes presented with weakness, headache, cognitive decline, and neurological deficits. Neurological examination revealed spastic paraparesis, cranial nerve involvement, and abnormal reflexes. In investigations, MRI highlighted white matter abnormalities. An oriented physiotherapy rehabilitation program was planned that mainly focused on neurological, cognitive, communication, balance, coordination, visual, and perceptual disturbances. In the rehabilitation program, task-specific training for weak muscles, errorless learning strategy for forgetfulness, speech-language therapy for slurred speech, facial muscle retraining exercises for improving facial muscle movements, balance and coordination exercises, VRT, vergence exercises for visual impairment, and pelvic floor strengthening exercises were found to be effective for the patient of leukoencephalopathy in the above case. The patient's progress was tracked using multiple outcome measures, including the Montreal Cognitive Assessment (MoCA), Barthel Index, and Functional Independence Measure (FIM). Pre-intervention scores indicated severe dependency, while post-intervention scores showed significant improvement, reflecting enhanced cognitive and functional abilities. The patient was advised to a home exercise program after six weeks of rehabilitation. The presented case underscores the importance of tailored rehabilitation protocols in addressing the complex symptoms associated with this condition.

## Conclusions

Patients with CSF1R-related leukoencephalopathy show significant challenges, and its management requires a multidisciplinary approach. The rehabilitation program that was planned for six weeks focused on various domains affected by leukoencephalopathy. Goal-oriented interventions including strength training, task-specific exercises, errorless learning strategies, speech-language therapy, facial muscle retraining exercises, and balance and coordination exercises were tailored to the patient's needs and reduce symptoms, improving functional capacity and enhancing the overall quality of life for affected individuals. Tailored rehabilitation programs, as demonstrated in this case, prove instrumental in addressing the complex and varied manifestations of this condition.

## References

[1] Filley CM, Kleinschmidt-DeMasters BK (2024). Toxic leukoencephalopathy. N Engl J Med.

[2] Bogousslavsky J, Regli F, Uske A (1987). Leukoencephalopathy in patients with ischemic stroke. Stroke.

[3] Shirah B, Algahtani H, Algahtani R, Alfares A, Hassan A (2023). Cerebral autosomal recessive arteriopathy with subcortical infarcts and leukoencephalopathy (CARASIL): a challenging diagnosis and a rare multiple sclerosis mimic. J Stroke Cerebrovasc Dis.

[4] Ikeuchi T, Mezaki N, Miura T (2018). Cognitive dysfunction and symptoms of movement disorders in adult-onset leukoencephalopathy with axonal spheroids and pigmented glia. Parkinsonism Relat Disord.

[5] Hinchey J, Chaves C, Appignani B (1996). A reversible posterior leukoencephalopathy syndrome. N Engl J Med.

[6] Renaud DL (2016). Adult-onset leukoencephalopathies. Continuum (Minneap Minn).

[7] Rowe F (2009). Visual perceptual consequences of stroke. Strabismus.

[8] Konno T, Kasanuki K, Ikeuchi T, Dickson DW, Wszolek ZK (2018). CSF1R-related leukoencephalopathy: a major player in primary microgliopathies. Neurology.

[9] Sundal C, Wszolek ZK (2012). CSF1R-related adult-onset leukoencephalopathy with axonal spheroids and pigmented glia. GeneReviews.

[10] Papapetropoulos S, Pontius A, Finger E (2021). Adult-onset leukoencephalopathy with axonal spheroids and pigmented glia: review of clinical manifestations as foundations for therapeutic development. Front Neurol.

[11] Shprecher D, Mehta L (2010). The syndrome of delayed post-hypoxic leukoencephalopathy. NeuroRehabilitation.

[12] Patten C, Lexell J, Brown HE (2004). Weakness and strength training in persons with poststroke hemiplegia: rationale, method, and efficacy. J Rehabil Res Dev.

[13] Middleton EL, Schwartz MF (2012). Errorless learning in cognitive rehabilitation: a critical review. Neuropsychol Rehabil.

[14] Koyuncu E, Çam P, Altınok N, Çallı DE, Duman TY, Özgirgin N (2016). Speech and language therapy for aphasia following subacute stroke. Neural Regen Res.

[15] Cronin GW, Steenerson RL (2003). The effectiveness of neuromuscular facial retraining combined with electromyography in facial paralysis rehabilitation. Otolaryngol Head Neck Surg.

[16] Hanna KL, Hepworth LR, Rowe FJ (2017). The treatment methods for post-stroke visual impairment: a systematic review. Brain Behav.

[17] Thomas LH, Coupe J, Cross LD, Tan AL, Watkins CL (2019). Interventions for treating urinary incontinence after stroke in adults. Cochrane Database Syst Rev.

[18] Kwon S, Koo J, Lee S (2001). Clinical spectrum of reversible posterior leukoencephalopathy syndrome. Pediatr Neurol.

[19] Ozyurek H, Oguz G, Ozen S, Akyuz C, Karli Oguz K, Anlar B, Aysun S (2005). Reversible posterior leukoencephalopathy syndrome: report of three cases. J Child Neurol.

[20] Liao YC, Hu YC, Chung CP, Wang YF, Guo YC, Tsai YS, Lee YC (2021). Intracerebral hemorrhage in cerebral autosomal dominant arteriopathy with subcortical infarcts and leukoencephalopathy: prevalence, clinical and neuroimaging features and risk factors. Stroke.

[21] Ameer MA, Bhutta BS, Asghar N, Haseeb MT, Abbasi RN (2021). Cerebral autosomal dominant arteriopathy with subcortical infarcts and leukoencephalopathy (CADASIL) presenting as migraine. Cureus.

[22] Katyal N, Narula N, George P, Nattanamai P, Newey CR, Beary JM (2018). Delayed post-hypoxic leukoencephalopathy: a case series and review of the literature. Cureus.

[23] PL F, PO JB, HA RF (2024). Delayed neurological deterioration after anoxia. Arch Intern Med.

